# Domestic Summary

**Published:** 1840-03-28

**Authors:** 


### DOMESTIC SUMMARY.

*Death of Dr. Allison.*—Dr. J. J. Allison, a young physician of Philadelphia, of great promise, died recently after a protracted illness. He was favourably known for industry and success in experimental physiological investigation, as well as for general professional attainments, and was highly esteemed for moral worth and amiability of character.

*Transylvania Medical School.*—The annual announcement for 1840, presents a list of sixty-two graduates, and two hundred and fifty-seven pupils—a larger class than those of many previous sessions, and one of the largest that has attended the school at Lexington. The valedictory address of Professor Mitchell, in his usual successful style, urges modestly, but forcibly, the many claims of Traansylvania upon the students of the West.

*Dr. Fletcher's Improved Truss.*—This truss, for some time in general use in New England, possesses modifications which are worth the notice of our southern surgeons. It is recommended by surgeons of eminence in the east, upon whose judgment reliance may be placed.

